# Cardiac Arrest during Gamete Release in Chum Salmon Regulated by the Parasympathetic Nerve System

**DOI:** 10.1371/journal.pone.0005993

**Published:** 2009-06-19

**Authors:** Yuya Makiguchi, Shinya Nagata, Takahito Kojima, Masaki Ichimura, Yoshifumi Konno, Hideki Murata, Hiroshi Ueda

**Affiliations:** 1 Division of Biosphere Science, Graduate School of Environmental Science, Hokkaido University, Sapporo, Hokkaido, Japan; 2 Dainippon Sumitomo Pharma Co. Ltd., Suita, Osaka, Japan; 3 College of Bioresource Sciences, Nihon University, Fujisawa, Kanagawa, Japan; 4 Shibetsu Salmon Museum, Shibetsu, Hokkaido, Japan; 5 Field Science Center for Northern Biosphere, Hokkaido University, Sapporo, Hokkaido, Japan; University of Arizona, United States of America

## Abstract

Cardiac arrest caused by startling stimuli, such as visual and vibration stimuli, has been reported in some animals and could be considered as an extraordinary case of bradycardia and defined as reversible missed heart beats. Variability of the heart rate is established as a balance between an autonomic system, namely cholinergic vagus inhibition, and excitatory adrenergic stimulation of neural and hormonal action in teleost. However, the cardiac arrest and its regulating nervous mechanism remain poorly understood. We show, by using electrocardiogram (ECG) data loggers, that cardiac arrest occurs in chum salmon (*Oncorhynchus keta*) at the moment of gamete release for 7.39±1.61 s in females and for 5.20±0.97 s in males. The increase in heart rate during spawning behavior relative to the background rate during the resting period suggests that cardiac arrest is a characteristic physiological phenomenon of the extraordinarily high heart rate during spawning behavior. The ECG morphological analysis showed a peaked and tall T-wave adjacent to the cardiac arrest, indicating an increase in potassium permeability in cardiac muscle cells, which would function to retard the cardiac action potential. Pharmacological studies showed that the cardiac arrest was abolished by injection of atropine, a muscarinic receptor antagonist, revealing that the cardiac arrest is a reflex response of the parasympathetic nerve system, although injection of sotalol, a *β*-adrenergic antagonist, did not affect the cardiac arrest. We conclude that cardiac arrest during gamete release in spawning release in spawning chum salmon is a physiological reflex response controlled by the parasympathetic nervous system. This cardiac arrest represents a response to the gaping behavior that occurs at the moment of gamete release.

## Introduction

**Figure 1:**
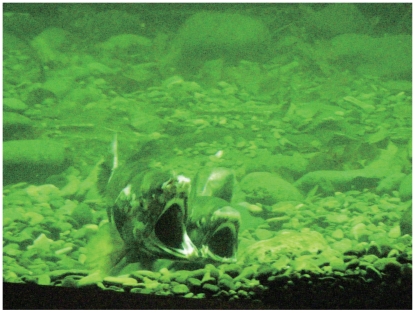
Gamete release in chum salmon. A pair of chum salmon tagged with electrocardiogram (ECG) data loggers, spawning in the Shibetsu Salmon Museum in Hokkaido, Japan. The fish on the left is male; the one on the right is female.

Animals have a sophisticated cardiovascular system, which is regulated by the central nervous system, to optimize their aerobic metabolism in response to internal and external changes [1]. Previous studies have reported that startling stimuli, such as visual and vibration stimuli, decrease ventilation and heart rate temporarily (bradycardia) and can lead to cardiac arrest in some animals including molluscs [2], crustaceans [3], fish [4], amphibians [5] and mammal [6]. This cardiac arrest could be considered as an extraordinary case of bradycardia and defined as reversible missed heart beats. Some researchers have interpreted the cardiac arrest as an adaptation for predator avoidance that reduces movement and noise from that animal [4], [7], [8]. In addition, variability of the heart rate is controlled by a balance between cholinergic vagus inhibition and excitatory adrenergic stimulation of neural and hormonal action [9], suggesting that regulation of the temporal cardiac arrest may be under the control of autonomic systems. Furthermore, cardiac arrest has been reported to occur for several seconds at the moment when the female releases eggs and male ejaculates sperm in the teleost chum salmon *Oncorhynchus keta* [10] that showed increased heart rate of the fish around the cardiac arrest from the usual rate. The authors observed electrocardiogram of chum salmon during spawning behavior by using a radio telemetry system in combination with a wired system from a pair of fish, and reported that the cardiac arrest might be a reflex response of the cardiovascular to the elevated blood pressure at the moment of gamete release in chum salmon. However, the cardiac arrest and the mechanism that regulates it remain poorly understood. Moreover, at the moment of gamete release in spawning chum salmon, female and male fully gape for several seconds. However, a physiological relationship between the gaping behavior and the cardiac arrest at the moment of gamete release is also unclear. Here we have monitored the cardiac arrest in spawning chum salmon (Fig. 1) with electrocardiogram (ECG) data loggers, and we show that this cardiac arrest is regulated by the parasympathetic nerve system.

## Results

**Figure 2:**
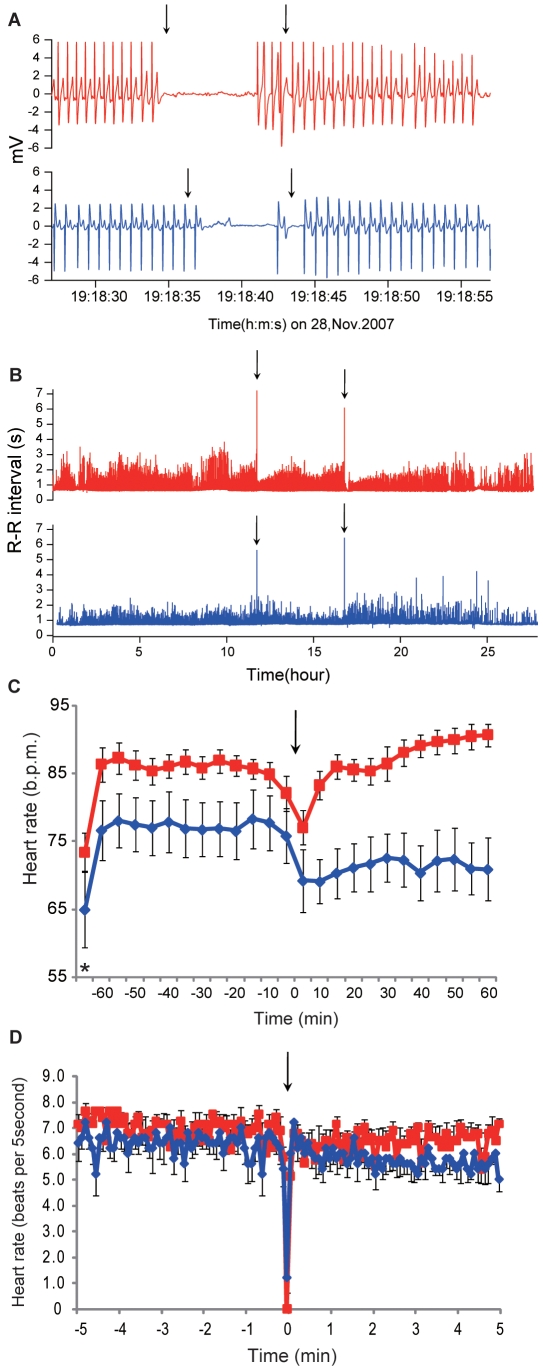
Cardiac arrest and heart rate changes during spawning behavior in chum salmon. (A) Electrocardiogram (ECG) at the moment of gamete release in female (red line) and male (blue line) chum salmon. Vertical arrows indicate the start and end point when fish open their mouth (gaping). (B) Time-series plots of the R-R interval of female (red line) and male (blue line) chum salmon during the spawning behavior. Vertical arrows show the first and second spawning, respectively. The averaged heart rate (b.p.m.) of the female and male individual is 85.8 and 76.7 respectively. (C) Time-series plots of the heart rate (b.p.m.) of female (red line, *n* = 8) and male (blue line, *n* = 5) chum salmon during the spawning behavior an hour before and after gamete release. Vertical arrow indicates spawning. Asterisk shows the heart rate during the resting period before the spawning behavior started. (D) Time-series plots of the heart beats for every 5 second period of female (red line, *n* = 8) and male (blue line, *n* = 5) chum salmon during the spawning behavior 5 minute before and after gamete release. Vertical arrow indicates spawning.

All tagged fish (eight females and five males) spawned once or twice each. Fifteen instances of egg release in females and ten instances of sperm ejaculation in males were observed, and twenty-five ECG signals during spawning behavior were recorded in total. Cardiac arrest occurred at the moment of gamete release in all fish and lasted for 4.93–10.02 s (6.81±0.54 s, *n* = 8) in females and for 3.41–6.51 s (4.81±0.60 s, *n* = 5) in males in the first spawning, and for 5.87–10.88 s (7.80±0.72 s, *n* = 7) in female and for 4.97–6.45 s (5.49±0.30 s, *n* = 5) in males in the second spawning (Fig. 2A). The difference between males and females in the duration of the cardiac arrest was significant for both the first and second spawning (Students *t*-test, *P*<0.01, respectively). The beginning of the cardiac arrest was synchronized with opening of the mouth (gaping) at the moment of gamete release. Furthermore, such a long duration of cardiac arrest was observed only at the moment of gamete release (Fig. 2B).

Throughout the spawning behavior, the heart rate was relatively higher in females than in males. The heart rate of the fish increased from an hour before the spawning behavior started until the fish finished releasing gametes (73.4±2.9 b.p.m. in female, *n* = 8 and 65.0±5.6 b.p.m. in male, *n* = 5 during the resting period). The fish showed an escalated heart rate just prior to spawning (86.2±1.5 b.p.m. in female, *n* = 8 and 76.6±4.2 b.p.m. in male, *n* = 5), but the heart rate decreased to 10.6±5.6% (77.1±2.5 b.p.m., *n* = 8) in females and 9.7±4.8% (69.2±4.6 b.p.m., *n* = 5) in males at the moment of gamete release (Fig. 2C). The heart rate calculating beats for every 5 second period clearly showed the sharp decreasing beats at the moment of gamete release for both sexes (Fig. 2D). The heart rate remained high after spawning only in females, demonstrating clear a sex difference in the spawning behavior of salmonids (Fig. 2C). Females built the nest using a caudal fin (“nest digging”) [11], a behavior that requires higher energy in females than in males during spawning behavior [12].

**Figure 3:**
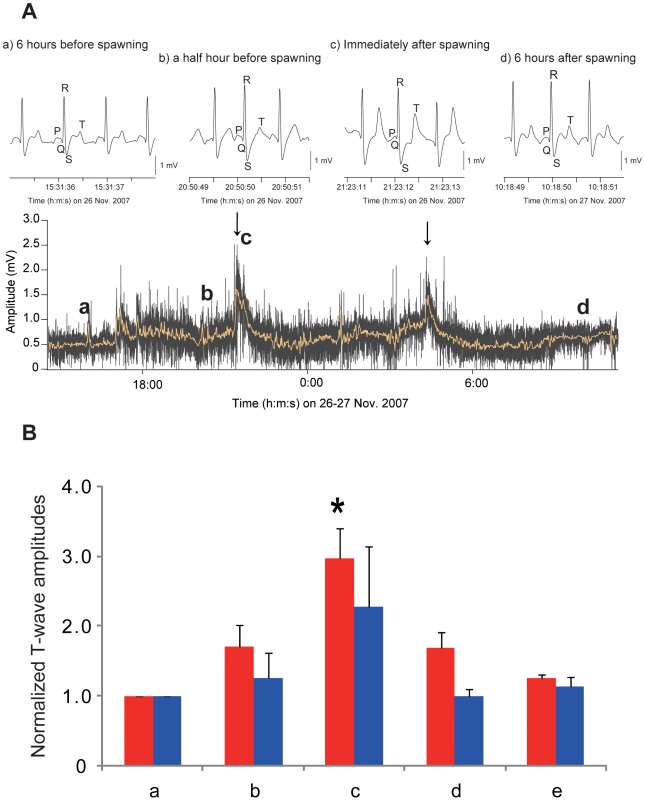
ECG morphology for time-series plots of T-wave amplitude in spawning female chum salmon. (A) Vertical arrows indicate the first and second spawning. The amplitude of T-waves gradually increases as the release of eggs approaches 6 hours before spawning (a) and a half-hour before spawning (b); a peaked and tall T-wave is found immediately after spawning (c); the amplitude then returns to pre-spawning levels 6 hours after spawning (d). (B) A histogram of normalized T-wave amplitude of female (red bars, *n* = 6) and male (blue bars, *n* = 4) at 6 hours before spawning (a), a half-hour before spawning (b), immediately after spawning (c), a half-hour after spawning (d) and 6 hours after spawning (e). Statistical analysis was performed by ANOVA with Dunnett's multiple comparison of mean test. Asterisk shows a significant difference compared with the normalized T-wave amplitude at 6 hours before spawning.

ECG morphology for the T-wave amplitude was calculated as the average of ten consecutive T-wave amplitudes that were normalized by the baseline T-wave amplitude (ECG signals measured approximately 6 hours before spawning). ECG morphological analysis showed that the T-wave amplitude gradually increased as spawning behavior became more advanced, and it peaked at the moment of gamete release and returned to the baseline levels approximately 6 hours after spawning (Fig. 3A) and this trend was found in both sexes (Fig. 3B). A significant elevation in the normalized T-wave at the moment of gamete release was observed at the first (3.03±0.41in female, *n* = 6 and 2.29±0.86 in male, *n* = 4) and second (4.17±1.46 in female, *n* = 5 and 2.49±0.92in male, *n* = 4) spawning in both sexes (Welch's *t*-test, *P*<0.05 for both sexes), and the T-wave amplitude tended to be higher in females than in males (Welch's *t*-test, *P* = 0.21).

**Figure 4:**
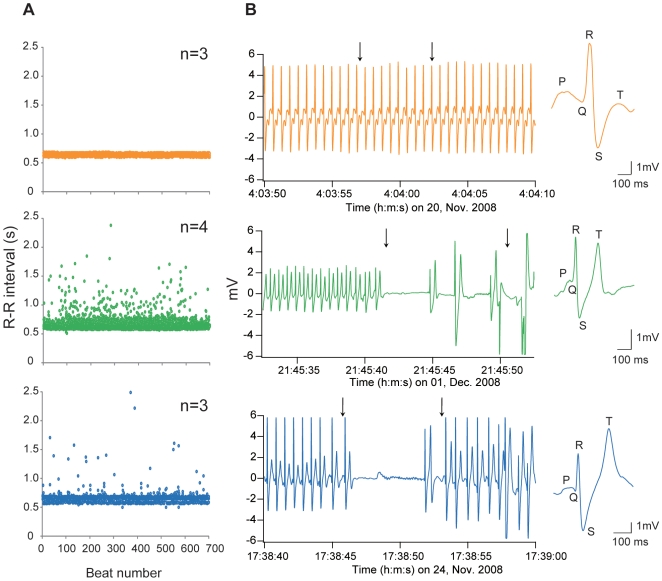
Effects of pharmacological injections on R-R interval, cardiac arrest, and ECG morphology in female salmon. (A) Tachogram of 700 consecutive heart beats immediately after egg release in fish injected with atropine (orange circles, *n* = 3), sotalol (green circles, *n* = 4), and Salmon Ringer as a sham control (blue circles, *n* = 3). (B) ECG at the moment of egg release in fish injected with atropine (orange line), sotalol (green line), and Salmon Ringer as a sham control (blue line). Vertical arrows indicate the start and end points of gaping. At the moment of egg release, peaked and tall T-waves occur in fish injected with sotalol and Salmon Ringer, but not in fish injected with atropine.

All females that were injected with pharmacological autonomic antagonists and monitored with ECG data loggers spawned between one and three times each, and the ECG signals during eighteen instances of egg release were recorded in total (Movie S1 for the fish injected with Salmon Ringer solution and Movie S2 for the fish injected with atropine). Each fish spawned from one to three times. Thus, we pooled the data from each individual in each group. The effects of sotalol on the heart rate (the R-R intervals) were apparent, with a reduction in the heart rate of approximately 29.6% (66.9±5.5 b.p.m.) as compared with control fish (95.1±0.7 b.p.m.) an hour after the injection. The heart rate was unaffected by atropine treatment (94.2±3.4 b.p.m.) as compared with control fish. After the spawning behavior had finished, the heart rate of fish injected with sotalol was reduced by 2.0% (89.8±1.3 b.p.m.) as compared with the heart rate of control fish (91.1±3.1 b.p.m.), and the heart rate of fish injected with atropine (92.1±2.1 b.p.m.) was similar to that of control fish. However, atropine treatment abolished the variability of the R-R intervals after the spawning behavior had finished (compared with the heart rate in control fish, *F*-test *P*<0.01, Fig. 4A). Therefore, we assumed that the effects of atropine injection on heart rate were maintained consistently until the spawning behavior finished, whereas the effects of sotalol injection might be attenuated. The elapsed time between data-logger attachment and the spawning episodes were 28.9±10.0 hours (16.4–58.9 hours, *n* = 4) in fish injected with sotalol. Cardiac arrest occurred at the moment of egg release in all fish injected with sotalol (4.7±1.2 s, *n* = 4) and in the control fish (5.6±1.1 s, *n* = 8, Fig. 4B). However, cardiac arrest was not observed in all 3 fish injected with atropine despite confirmation of egg release during the spawning behavior; thus, atropine injection abolished the cardiac arrest while the female released eggs. From the ECG morphological analysis, a significant increase in T-wave amplitude at the moment of egg release was found in fish injected with sotalol (*n* = 7) and in control fish (*n* = 8; Welch's *t*-test, *P*<0.05 for both groups). By contrast, this prominent T-wave was not observed in fish injected with atropine at the moment of egg release (Welch's *t*-test, *P* = 0.153; Fig. 4B).

## Discussion

This study revealed that a cardiac arrest lasting for approximately 7 s in females and 5 s in males occurred at the climactic moment when females released eggs and males ejaculated sperm, indicating that cardiac arrest is a characteristic physiological phenomenon in spawning chum salmon with a significant difference in its duration between the sexes. Contrary to the cardiac arrest previously reported in some animals that is the result of an external (startling) stimulation, the cardiac arrest that occurred during gamete release in chum salmon was the result of an internal stimulation. A cardiac arrest lasting a few seconds during sperm ejaculation has also been reported in male octopus *Octopus vulgaris* [13]. Although the biological meaning of the cardiac arrest in some animals remains unclear, cardiac arrest may not be unusual phenomenon during gamete release in some aquatic animals.

The ECG morphological analysis revealed that peaked and tall T-waves occurred adjacent to gamete release. A T-wave represents the period of ventricular repolarization. A prominent T-wave is an abnormal T-wave morphology that is encountered during acute myocardial infarction in humans, and an increase in serum potassium level frequently causes the T-wave trend to become tall and peaked [14]. Furthermore, this study showed that cardiac arrest did not occur during egg release in fish injected with atropine, a muscarinic receptor antagonist, indicating that this cardiac arrest is mediated by the parasympathetic nerve system. Activated parasympathetic neurons release the neurotransmitter, acetylcholine (ACh), which increases potassium permeability in cardiac muscle cells, and the higher potassium efflux retards the cardiac action potential towards the threshold for triggering an action potential, resulting in an extension of heart rate [15]. Vagus stimulation causes an increase in T-wave amplitude [16], and injection of ACh causes an increase in T-wave amplitude, a decrease in heart rate and missing beats (cardiac arrest) in dogs [17], [18]. Thus, we speculate that the cardiac arrest that occurs during gamete release is a reflex response to vagal cholinergic drive (parasympathetic activation). In addition, this study showed that cardiac arrest at the moment of gamete release was observed in fish injected with sotalol. Regulation of heart rate and its variability in short-horned sculpin *Myoxocephalus scorpius* is under parasympathetic, cholinergic control [19]. Thus, we speculated that chum salmon during spawning behavior might have a dominant cholinergic tone although the effects of sotalol injection might attenuate at the moment of gamete release. Here, we proposed the hypothesis that the cardiac arrest at the moment of gamete release is a physiological response to the behavioral response of gaping, which may cause a reduction in water flow over the gill. For teleost fish, the initial cardiac response to aquatic hypoxia is reflex bradycardia [20], [21], which is mediated by vagal cardio-inhibitory fibres [22]. The occurrence of increased systemic blood pressure accompanying the hypoxic bradycardia serves to open perfused vascular spaces in the gill lamellae, creating a more event blood flow within them, and recruiting unperfused lamellae to increase the effective area for gas exchange [23], [24]. In addition, the vasoactive mechanism also greatly affects the gill lamellar perfusion patterns [25]. Reflex cholinergic vasoconstriction in the vicinity of the gill filament arteries [26] is thought to enhance lamellar perfusion and oxygen uptake across the gills [27].

The fish showed an escalated heart rate during the spawning behavior as compared with the resting period in both sexes although the resting heart rate might be relatively high because of the handling stress of the attachment surgery. Energy expenditure during spawning behavior in salmon is relatively higher than standard metabolism [12]; as a result, spawning behavior represents relatively severe exercise. Therefore, we speculated that chum salmon greatly increased cardiac output to support increased metabolism during spawning behavior, because fish heart has a remarkable ability to produce large increases in cardiac stroke volume [22].

In vertebrates, baroreflex is essential in arterial pressure homeostasis, and fish has baroreceptor sites in the gills [28], [29]. Atropine administration abolishes the baroreflex response in fish, indicating that the origin of the reflex response that mediates modulation of heart rate is cholinergic [30]. In contrast to hypotension with tachycardia, teleost fish rapidly respond to increases in arterial blood pressure with vagus-mediated bradycardia [9]. Furthermore, salmon show a strong burst of activity of the trunk musculature at the moment of gamete release [31]. Taking all these data into consideration, the highest blood pressure resulting from transient hypoxia caused by gaping and the pressure of pushing out gametes might occur in the blood vessels at the moment of egg or sperm release and the cardiac arrest could be considered as an extraordinary case of bradycardia. In addition, cholinergic nerves directly innervate systemic blood vessels in the gill and the chromaffin cells, which are also localized in the heart and along the cardinal vein, and which produce catecholamine [9], [32]. In conclusion, we speculate that the cardiac arrest that occurs in spawning chum salmon when female release eggs and males ejaculate sperm represents a remarkable behavioral response of gaping under vagal cholinergic regulation.

## Materials and Methods

### Attachment procedure of ECG data logger

This study (No. 18–3) has been carried out under the control of the committee along the “Guide for the Care and Use of Laboratory Animals in Field Science Center for Northern Biosphere, Hokkaido University” and Japanese Governmental Law (No. 105) and Notification (No. 6). Eight female (61.5±7.7 cm fork length (*L*_F_), 2.6±0.08 kg mass) and five male (65.5±1.1 cm *L*_F_, 3.0±0.1 kg mass) chum salmon were tagged with an ECG logger (W400L-ECG, 21 mm in diameter, 110 mm in length, 57 g in air; Little Leonard Co., Tokyo, Japan) to record the heart rate as previously described [33] during 11–29 November 2007. In brief, chum salmon captured in the Shibetsu River estuary were anaesthetized using FA 100 (eugenol; Tanabe Seiyaku Co. Ltd, Osaka, Japan) at a concentration of 0.5 mL L^−1^. A bipolar electrode made by a copper disc (approximately 1.5 cm in diameter) was surgically attached on the ventral side by using sutures. The ECG loggers, sutured using nylon ties and instant glue (α-cyanoacrylate; Fujiwara Sangyo Co. Ltd, Hyogo, Japan), were tagged on the back of the fish, anterior to the dorsal fin, through small holes using two stainless needles. During the tagging procedure, which took approximately 20 min, the gills of the fish were irrigated with water containing diluted FA 100 to maintain sedation. The sampling rate of the ECG loggers was set at 200 Hz. After 24 hours to allow for recovery from the tagging, the spawning behavior of the fish was monitored with a digital video camera to synchronize recordings of behavior with the ECG signals in the spawning channel (3.8×2.9×1.1 m) connected to the Shibetsu River supplied with spring water (16.8°C) underneath a gravel bottom, which was free of silt [34], in the Shibetsu Salmon Museum, Hokkaido, Japan.

### Pharmacological study

For the injection of pharmacological autonomic antagonists, only females were tagged with an ECG logger during 3–29 November 2008. After the tagging procedure described above, the dorsal aorta was temporarily cannulated by using polyethylene tubing with a diameter of 1.3 mm via the upper jaw [34]. The fish were injected with atropine (a muscarinic antagonist; atropine sulfate, 1.2 mg/kg, SIGMA, Missouri, USA; 66.5±2.9 cm *L*_F_, 3.2±0.5 kg mass, *n* = 3) or sotalol (a *β*-adrenergic antagonist; sotalol hydrochloride, 2.7 mg/kg, SIGMA; 62.4±0.6 cm *L*_F_, 2.8±0.1 kg mass, *n* = 4) or 1 ml of Salmon Ringer solution (150 mM NaCl, 310 mM KCl, 0.40 mM HEPES; 4-(2-hydroxyethyl)-1-piperazineethanesulfonic acid, 0.34 mM CaCl_2_, 0.10 mM MgCl_2_, 0.03 mM MgSO_4_ with distilled water in total 1 L) as a sham control (64.9±1.9 cm *L*_F_, 3.0±0.4 kg mass, *n* = 3). Atropine and sotalol were dissolved in 1 ml of Salmon Ringer solution. After the injections, the spawning behavior of the fish was monitored as described above without any recovery period.

### Data analysis

Igor Pro (WaveMetrics Inc., Lake Oswego, OR, USA) and Fluclet WT (Dainippon Sumitomo Pharmacy Co., Ltd., Osaka, Japan) were used to determine the ECG intervals (R-R intervals) and morphology. Statistical significance was achieved at *P*<0.05. Values are presented as mean±standard error of the mean (s.e.m.). Heart rate is presented as beats per minute (b.p.m.).

## Supporting Information

Movie S1 This movie shows the spawning behavior and gamete release of chum salmon. The female was attached with an electrocardiogram (ECG) data logger and injected with Salmon Ringer solution (QuickTime; 1.2 MB).
(1.23 MB MOV)

Movie S2 This movie shows the spawning behavior and gamete release of chum salmon. The female was attached with an electrocardiogram (ECG) data logger and injected with atropine (QuickTime; 1.3 MB).
(1.31 MB MOV)
